# The effect on shikimate production by deleting *iolR* and metabolic engineering in PTS deficient *Corynebacterium glutamicum* strain

**DOI:** 10.3389/fbioe.2025.1616558

**Published:** 2025-06-26

**Authors:** Rui Wen Ou, Charles A. Swofford, En-Ze Linda Zhong-Johnson, Cheng Li, Anthony J. Sinskey

**Affiliations:** ^1^ Department of Biology, Massachusetts Institute of Technology, Cambridge, MA, United States; ^2^ Center for Biomedical Innovation, Massachusetts Institute of Technology, Cambridge, MA, United States

**Keywords:** shikimate, PTS deficient, *iolR*, *Corynebacterium glutamicum*, metabolic engineering

## Abstract

Shikimate is a precursor to many high-value chemical derivatives. Several bacteria have been engineered to produce high titer of shikimate *via* non-phosphotransferase system (Non-PTS), but yet explores how the myo-inositol utilization transcription regulator (*iolR*) deletion affects the shikimate titer in phosphotransferase system (PTS) deficient strain. In this study, we engineered *Corynebacterium glutamicum* to produce shikimate in a PTS deficient strain with the deletion of *iolR* and improved shikimate production using a metabolic engineering approach. PTS was eliminated to improve phosphoenolpyruvate levels, however, both the cell growth rate and shikimate production were dramatically reduced. Hence, *iolR* was deleted to improve cell growth and shikimate production in the PTS deficient strain. In addition, we overexpressed genes in the glycolysis and shikimate pathways to increase shikimate production. The combination of the strategies resulted in a shikimate content of 0.76 mg/mg of DCW and a titer of 4.1 g/L in shake flask in C. *glutamicum*, providing novel insights for further engineering to enhance production of shikimate and its derivatives.

## 1 Introduction

**FIGURE 1:**
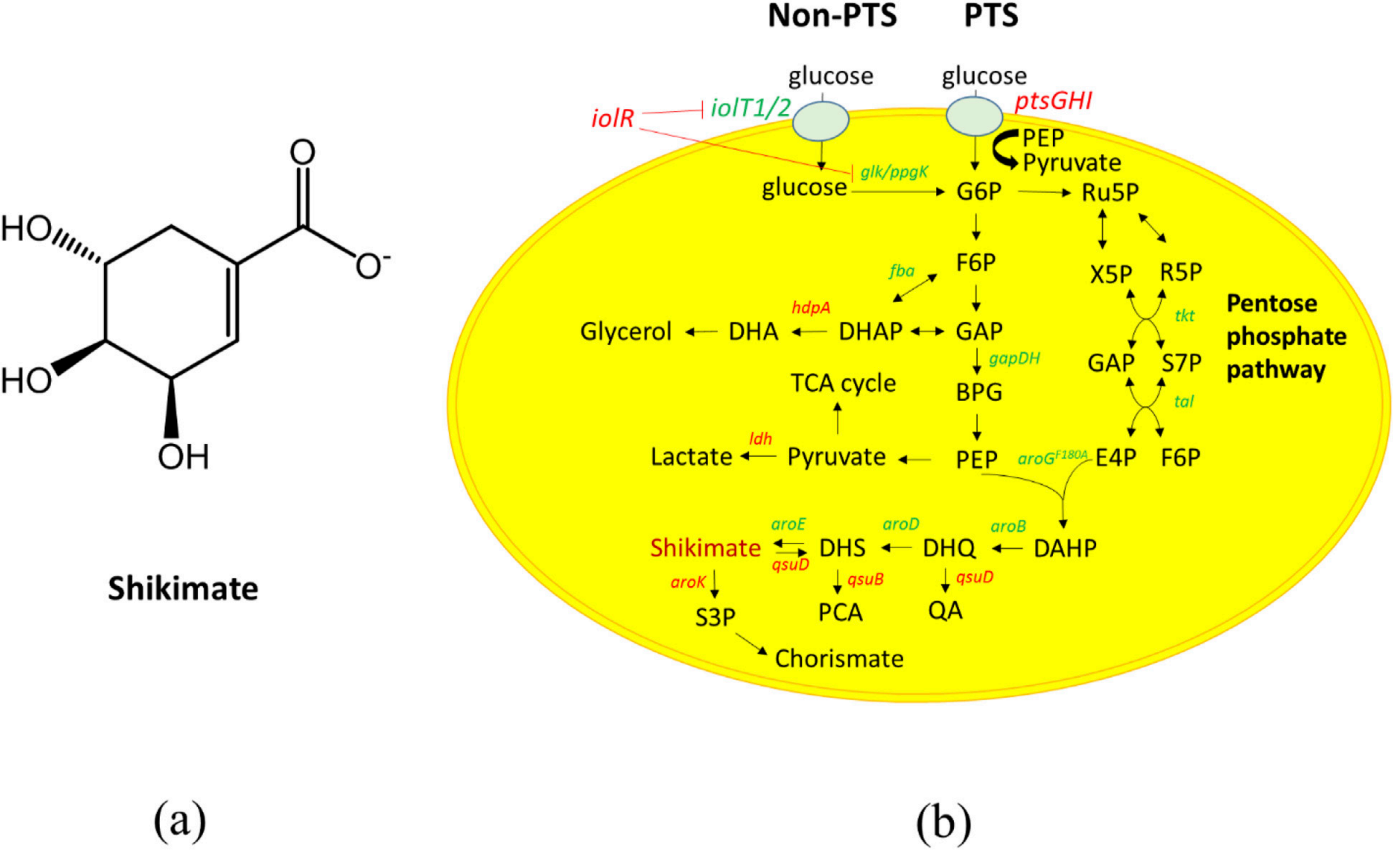
The biosynthesis of shikimate. **(a)** Chemical structure of shikimate, it has three chiral centers **(b)** Metabolic pathway of shikimate biosynthesis in *Corynebacterium glutamicum*. Black arrows represent the carbon flux. The red genes are deleted. The green genes are overexpressed. Dashed arrows represent two or more catalytic steps. Genes encoding enzymes for corresponding catalytic steps are indicated in italics. G6P, glucose-6-phosphate; F6P, fructose-6-phosphate; GAP, glyceraldehyde-3-phosphate; DHAP, 1,3-dihydroxyacetone phosphate; DHA, 1,3-dihydroxyacetone; BPG, 1,3-bisphosphoglycerate; PEP, phosphoenolpyruvate; PYR, pyruvate; AcCoA, acetyl-coenzyme A; OAA, oxaloacetate; CIT, citrate; Ru5P, ribulose-5-phosphate; R5P, ribose-5- phosphate; X5P, xylulose-5-phosphate; S7P, sedoheptulose-7-phosphate; E4P, erythrose 4-phosphate; DAHP, 3-deoxy-D-arabinoheptulosonate-7-phosphate; DHQ, 3-dehydroquinate; DHS, 3-dehydroshikimate; PCA, protocatechuate; S3P, shikimate-3-phosphate. Genes and coded enzymes: *ptsGHI*, phosphoenolpyruvate phosphotransferase consisting of *ptsG*, *ptsH*, and *ptsI*.; *iolT1*, myo-inositol permease; *glk*, glucokinase; *ppgk*, polyphosphate glucokinase; *iolR*: myo-inositol utilization transcriptional regulator; *tkt*, transketolase*; tal*, transaldolase; *gapA*, GAP dehydrogenase; *hdpA*, DHAP phosphatase; *aroG* ^*S180F*^, DAHP synthase with amino acid point mutation S180F from *E. coli*; *aroB*, DHQ synthase; *aroD*, DHQ dehydratase; *aroE*, shikimate dehydrogenase; *aroK*, shikimate kinase; *qsuB*, DHS dehydratase; *qsuD,* QA/shikimate dehydrogenase.

Shikimate (3,4,5-tri-hydroxy-1-cyclohexene-1-carboxylic acid) is a valuable compound that has been used as the starting material for the synthesis of Tamiflu (anti-viral symptoms suppressing drug) (Rawat et al., 2013). It is a metabolic intermediate found in the shikimate pathway as an aromatic amino acid precursor in microbes and plants, and is a precursor for many high-value derivatives (Tamiflu, caffeic acid, vitamin b9, vitamin K1/K2, vitamin E, flavonoids and others) for various applications in industry (Herrmann, 1995; Kallscheuer et al., 2017; Kramer et al., 2003; Pittard, 1996; Sato et al., 2001; Tzin and Galili, 2010; Wang et al., 2017). Shikimate has three chiral carbon centers which makes it difficult to synthesize chemically (Figure 1a). While most of the current shikimate supply is extracted from the fruit of the Chinese star anise (*Illicium verum*), limited raw material, low yield and costly extraction of shikimate have made it difficult to meet worldwide demand (Bochkov et al., 2012; Ghosh et al., 2012).

Recently, researchers have engineered microorganisms such as *Escherichia coli* and *C. glutamicum* R to produce 126.4 g/L and 141 g/L of shikimate, respectively (Kogure et al., 2016; Z. Li et al., 2023). In *Corynebacterium glutamicum* R shikimate producing strain, they utilized the non-PTS pathway to increase PEP precursor for shikimate production. However, the inactivation of PTS causes growth defects, and an alternative pathway is required to restore cell growth (Flores et al., 2005). Potential strategies to recover cell growth include the alternative glucose-facilitated diffusion transport (*glf*) from *Z. mobilis* with an additional copy of the glucokinase from *Zymomonas mobilis* or constitutively expressed the endogenous non-PTS glucose uptake route in *C. glutamicum* (which consists of the myo-inositol transporter *iolT1* and three glucokinases *glk1*, *glk2* and *ppgk*) (Chandran et al., 2003; Kogure et al., 2016; Li et al., 2023). Besides these two strategies, the deletion of the myo-inositol utilization transcriptional regulator (*iolR*) has also shown to recover cell growth and improve succinic acid production in a PTS deficient *C. glutamicum* strain (Zhou, et al., 2015). The effects of *iolR* deletion in *C. glutamicum* on cell growth and shikimate production remains unexplored.

Here, we used *C*. *glutamicum* (generally recognized as safe, gram-positive soil bacteria) to produce shikimate due to its ability to produce high amounts of amino acids (Park et al., 2014; Yokota and Ikeda, 2017). *Corynebacterium glutamicum* is a member of the Actinobacteria. It is a soil inhabiting, nonpathogenic microorganism. The benefits of using this microorganism are that it can grow fast on simple, chemically defined media and the genome sequence is well known (Becker and Wittmann, 2012; Ikeda, 2013; Kalinowski et al., 2003; Loos et al., 2001; Wendisch et al, 2006; Wieschalka et al., 2013). This microorganism has been used in industrial applications to produce amino acids, such as L-glutamic acid, L-lysine etc. An engineered *C. glutamicum R* strain was able to produce 141 g/L of shikimate in high cell density growth arrest fermentation (Kogure et al., 2016). However, different options to restore glucose up-take via the non-PTS pathway and their respective effects on shikimate production were not fully explored. This led us to construct a shikimate overproduction strain that utilizes an alternative strategy for glucose up-take in the non-PTS strain, and further use it as a platform to produce high value downstream products. In this study, we first eliminated by-products to produce shikimate. Next, we improved phosphoenolpyruvate (PEP) availability by deleting the phosphocarrier protein HPr (*ptsH*) to create a phosphoenolpyruvate-dependent PTS deficient strain. *iolR* was deleted to restore cell growth and improve shikimate production in the PTS deficient strain. Finally, the shikimate metabolic pathway was improved to enhance shikimate production.

## 2 Materials and methods

### 2.1 Bacterial strains and plasmids

The construction of different strains presented in this work were based on parental strain *C. glutamicum* ATCC 13032 from Bielefeld University, and *E. coli* TOP10F’ (Invitrogen, United States) was used as a host for plasmids construction. The pK18mobsacB suicide vector (Schafer et al., 1994) was used for all gene deletions and insertions in *C. glutamicum* via double crossover (Plassmeier et al., 2016). The shuttle vector pZ8-1 (Dusch et al., 1999) was used to introduce the *fba and GapDH* from *Elaeis guineensis* into *C. glutamicum*. All the strains and plasmids used in the present work are listed in Table 1; Supplementary Table S1, respectively.

**TABLE 1:** Bacterial strains and plasmids used in this study.

Strains	Characteristics	Source
*E. coli* TOP 10 F′	*F'{lacIqTn10(TetR)} mcrA Δ(mrr-hsdRMS-mcrBC) φ80lacZΔM15 ΔlacX74 recA1 araD139 Δ(ara-leu)7,697 galU galK rpsL endA1 nupG*	Invitrogen
*C. glutamicum* ATCC 13032	Wild-type	Bielefeld
CR100	*C. glutamicum* ATCC 13203 with the deletions of *CPG1/2/3, ISCg1c/d/a/e/b, ISCg2b/c/d/e/f, ISCg5a/b/c, ISCg13a-ISCg21a, ISCg13b, ISCg16a/b, ISCg8, ISCg12, ISCg3a/b*	Li et al. (2021)
S1	*C. glutamicum* MIT50 with deletions of *aroK* and *qsuD*	This work
S2	S1 with deletion of *qsuB*	This work
S3	S2 with deletion of *ldh*	This work
S4	S3 with deletion of *hdpA*	This work
S4.5	S4 *with deletion of ptsH*	This work
S5	S4 with deletion of *ptsH* and *iolR*	This work
S6	S5 with deletion of *aroG* and integrated overexpressed *aroG* ^*S180F*^ * E. coli* under a trc promoter	This work
S7	S6 with an integrated of trc promoter to overexpress *tkt* and *tal* operon	This work
S8	S7 with an integrated of trc promoter to overexpress *iolT1*	This work
S9	S8 with an integrated of *aroD, aroE* and *aroB*_*C. efficiens*_ overexpressed under trc promoter replaced *ldh*	This work
S10	S9 with integration of *glk* and *ppgk* from *C. efficiens* after the overexpressed iolT1 under trc promoter	This work
S10.5	S10 with a pZ8 plasmid containing overexpressed *fba* and *gapDH* from *Elaeis guineensis* under a *tac* promoter	This work
S11	S10 with an integrated of tac promoter to overexpress *fba* and *gapDH* from *Elaeis guineensis*	This work

### 2.2 Plasmid and strain construction

All cloning techniques used in this study (PCR amplification, purification, digestion, and electroporation) were performed according to protocols previously described (Russell and Sambrook, 2001). The ligation was done using HiFi 2X Assembly mix (New England Biolabs). PfuUltra High-Fidelity DNA Polymerase (New England Biolabs) was used to amplify PCR products for cloning. Taq 2X Master Mix (New England Biolabs) was used for amplifying PCR products for DNA sequencing (Quintara Biosciences). All primers (IDT) are listed in Supplementary Table S2. All codon optimized genes (Thermo Fisher Scientific) are listed in Supplementary Table S3. The Zymo Miniprep Kit (Zymo), Zymo Gel Extraction Kit (Zymo) and Zymo PCR Purification Kit (Zymo) were used to purify the plasmids, extract PCR products and purify PCR products, respectively. Preparation of the competent *C. glutamicum* cells and electroporation were done as previously described (Dunican, 1989; van der Rest et al., 1999).

The gene deletions and integrations in the *C. glutamicum* chromosome were performed using the suicide vector pK18mobsacB. The PCR product of the pK18mobsacB and DNA insert, amplified with the primers given in Supplementary Table S2, were ligated by Gibson Assembly method (Gibson et al., 2009), and then introduced into *E. coli TOP10 F′* competent cells using a heat shock method (Froger and Hall, 2007). The correct clones were selected according to PCR colony sequencing. The expression vector pZ8-1 (Dusch et al., 1999) was used to overexpress codon optimized genes (listed in Supplementary Table S3) in *C. glutamicum*. The overexpression plasmid was constructed using the primers listed in Supplementary Table S2 and transformed into *E. coli*, and plasmid construct containing the corrected genes were transformed into *C. glutamicum* strain. The overexpression of *fba* and *gapDH* from *E. guineensis* is done using the *P*_tac_ promoter.

### 2.3 Culture media and culturing conditions

*Escherichia coli* were grown in lysogeny broth (LB; Difco) or on LB agar plates containing kanamycin (50 mg/L) at 37° and *C. glutamicum* strains were grown in CASO broth (Sigma-Aldrich) or on CASO agar plates with/without kanamycin (25 mg/L) at 30°. The media used for preparation of *C. glutamicum* competent cells and transformation of plasmids into *C. glutamicum* competent cells were previously described (van der Rest et al., 1999). For plasmid and strain selection, kanamycin (Calibiochem, 50 mg/L for *E. coli*, 25 mg/L for *C. glutamicum*) were used. CASO agar containing 10% (w/v) sucrose (Sigma-Aldrich) was used to counter-select against SacB. *Corynebacterium glutamicum* strains for shikimate production were grown in CGXII (Keilhauer et al., 1993) defined minimal medium, containing the following per liter distilled water: 40 g glucose, 20 g (NH4)_2_SO4, 5 g urea, 1 g KH_2_PO_4_, 1 g K_2_HPO_4_, 0.25 g MgSO_4_ 7H_2_O, 42 g 3-morpholinopropanesulfonic acid, 10 mg CaCl_2_, 10 mg FeSO_4_ 7H_2_O, 10 mg MnSO_4_ H_2_O, 1 mg ZnSO_4_ 7H_2_O, 0.2 mg CuSO_4_, 0.02 mg NiCl_2_ 6H_2_O, 0.2 mg biotin, 0.5 mg thiamin, 30 mg protocatechuic acid, and addition of 100 mg/L of phenylalanine, 100 mg/L tyrosine, 100 mg/L tryptophan and 50 mg/L *p*-aminobenzoate. When appropriate, kanamycin (25 mg/L) was added. For shikimate production in shake flasks, the cells were grown at 30° overnight, then a single colony of cells was used to inoculate in 5 mL of CASO medium in test tube. Overnight cells in CASO medium were transferred to 25 mL of defined minimal medium at final cell optical density at 600 nm (OD_600_) of 0.3 as to initiate cell growth for shikimate production. The cells were grown at 30°C in a shaker incubator with 200 rpm agitation.

### 2.4 RNA purification and quantitative real-time PCR

Real-time quantitative PCR (qRT-PCR) was used to quantify the relative gene expression of *iolT1/2, glk* and *ppgk*. Total mRNA was extracted and purified by RNeasy Mini kit (Qiagen, United States) and was used as the template for cDNA library generation. cDNA libraries were prepared using SuperScript III First-Strand Synthesis System for RT-PCR (Invitrogen, United States). qRT-PCR was carried out on a CFX96™ Real-Time System (Bio-Rad, United States) using iTaq Universal SYBR Green Supermix (Bio-Rad, United States) according to the manufacturer’s instructions. *LeuA* was used as a reference gene for normalization (Wang et al., 2018). The relative normalized gene expression of *iolT1/2*, *glk*, and *ppgk* were calculated using Bio-Rad CFX Maestro data analysis software.

### 2.5 Analytical methods

Samples were centrifuged (15,000 g, 4° for 5 min) and concentrations of glucose and shikimate in the supernatants were determined. The quantification of glucose was performed on an Agilent 1,200 series HPLC system using an Aminex HPX-87H Ion Exclusion column and a refractive index detector. 1 mL of cell culture sample was centrifuged at 16,000 g for 4 min, and then a 1:10 dilution was prepared. The sample dilution was filtered through a 13 mm syringe filter with 0.2 μm PTFE membrane. 25 μL of sample was injected into the column with a flow rate of 0.6 mL/min at 50°C with 5 mM Sulfuric acid solution. The glucose standard curve was generated using glucose (Sigma-Aldrich, St. Luis, MO). Concentration of shikimate was determined by HPLC (Agilent 1,200 series) equipped with a C18 Reverse phase column (Agilent) operating at 40°C with a mobile phase of 20% methanol with 0.07% perchloric acid at a flow rate of 1.0 mL/min (Kogure et al., 2016). Shikimate concentration was measured at a wavelength of 210 nm. Optical density measurement at 600 nm were used to monitor cell growth, and three independent culture samples were measured for each growth curve. Data are averages and standard deviation error of the results from triplicates (* represent p-value <0.05 for two-tailed t-test). Shikimate standard curve was established on the HPLC using standard purchased from Sigma-Aldrich (Supplementary Figure S2).

Microscopy (Nikon Eclipse Ti-E) was used to observe the cells morphology at 100X with 5 μL of cell samples at 41, 72 and 120 h. Images were taken with the NIS-Elements AR3.2 software.

## 3 Results

### 3.1 Construction of a shikimate producing *Corynebacterium glutamicum* using a stable strain

**FIGURE 2:**
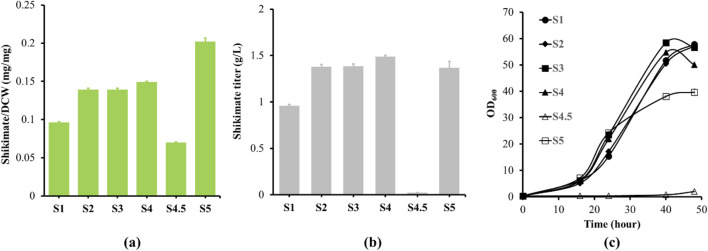
Shikimate and growth analysis from engineered strains (S1 to S5). **(a)** S1 to S5 strains were cultured in 250 mL shake flask with the define CGXII minimum medium supplemented with 4% glucose for 48 h. Shikimate contents (mg/mg DCW) were measured at 48 h, are labeled in green bar. **(b)** Shikimate titers (g/L) were measured at 48 h for S1 and S5. **(c)** To observe the phenotypes after gene deletions, strains were cultivated in the defined CGXII minimal medium with 4% glucose as the only source of carbon and energy. The cell growth curves of S1 (filled circle), S2 (filled diamond), S3 (filled square), S4 (filled triangle), S4.5 (opened triangle) and S5 (opened square) are shown. Data are averages and standard deviation error of the results from triplicates. OD_600_, optical density at 600 nm.

We utilized *C. glutamicum* strain CR100 as the parent strain for constructing a shikimate producing *C. glutamicum* strain, which has enhanced genetic stability and has ten operons of insertion sequence (IS) elements deleted from its chromosome (Li et al., 2021). *Corynebacterium glutamicum* can produce shikimate; however, it does not accumulate because it is a precursor to many downstream products (e.g., aromatic amino acids, protocatechuic acid, menaquinone, and others). To accumulate shikimate in *C. glutamicum*, we deleted *aroK* (shikimate to shikimate-3-phosphate) and *qsuD* (shikimate to 3-dehydroshikimate and 3-dehydroquinate to quinic acid) (Figure 1b) (Kubota et al., 2013; Teramoto et al., 2009). Deletion of *aroK* and *qsuD* results in auxotrophy for aromatic amino acids and *p*-aminobenzoate (Kogure et al., 2016). For the strain to grow, we added aromatic amino acids and *p*-aminobenzoate to the CGXII medium. As a result, the *aroK* and *qsuD* deletion strain (S1) was able to accumulate a shikimate content of 0.096 mg/mg and titer of 0.957 g/L in the culture media after 48 h (Figures 2a,b). The cell growth was able to reach OD_600_ of 55 after 48 h (Figure 2c).

### 3.2 Eliminating by-products formation to improve shikimate titer in *Corynebacterium glutamicum*

The shikimate pathway contains many intermediates and by-products such as protocatechuic acid, lactacte and glycerol, and elimination of these by-products will likely improveshikimate production (Figure 1b). We deleted DHS dehydratase (*qsuB*)*,* lactate dehydratase (*ldh*) and DHAP phosphatase (*hdpA*)*,* which are involved in the production of protocatechuic acid, lactic acid, and glycerol, respectively (Bunch et al., 1997; Hawkins et al., 1993; Jojima et al., 2012; Jojima et al., 2015) (Figure 1b). Deletion of *qsuB* (strain S2) resulted in 0.139 mg/mg of shikimate content and 1.37 g/L of shikimate titer in the culture medium after 48 h (Figures 2a,b). Then, *ldh* was deleted (strain S3), but no significant effect on shikimate production was observed. Finally, deletion of *hdpA* (strain S4) produced 0.149 mg/mg of shikimate content and 1.49 g/L of shikimate titer accumulated in the medium after 48 h. With these three deletions, shikimate content improved 1.5-fold, while none of the deletions affected growth rate (Figure 2c).

### 3.3 *iolR* deletion improved cell growth and shikimate production in PTS deficient strain

To further improve the shikimate titer, the availability of PEP was increased. PEP is an important precursor for shikimate production (Flores et al., 1996), but a large fraction is converted to pyruvate via the PTS system. Shin *et al.* showed that there are two transport systems for glucose into the cell (Shin et al., 2018). They are utilizing the PTS pathway or non-PTS pathway. The PTS pathway uses PEP as the phosphate donor to phosphorylate sugar across cell membrane, and converts PEP to pyruvate. In contrast, non-PTS pathway uses the myo-inositol transporter to transport glucose across cell membrane, then the glucose is phosphorylated to glucose-6-phosphate. Non-PTS pathway does not use PEP as the phosphate donor to phosphorylate sugars across cell membrane; therefore, it enhances PEP availability for shikimate production. Inactiviation of the PTS pathway should enhance PEP availiability and the presence of the non-PTS pathway should allow for glucose transport into the cell for growth and shimikate production. *PtsH*, a component of the PTS transport system, was deleted (strain S4.5) to enhance PEP availiability. However, the deletion of *ptsH* elongated the lag phase to 2 days while the other strains’ lag phase is less than 1 day (Figure 2c). As a result, 0.07 mg/mg of shikimate content and 0.023 g/L of shikimate titer accumulated in the medium after 48 h, equal to a 2.1-fold reduction in shikimate content compared to strain S4 (Figures 2a,b). When the lag phase ended, strain 4.5 entered an exponential growth phase (Supplementary Figure S2). PTS is therefore likely the preferred pathway for glucose uptake, and that glucose uptake through the non-PTS pathway is likely low or inhibited in *C. glutamicum* during the lag phase.

Zhou et al. previously demonstrated that the non-PTS pathway is inhibited by *iolR,* which is a negative inhibitor of the myo-inositol pathway. To overcome the inhibition effect on myo-inositol transporters (*iolT1/2*) and glucose kinases (*glk* and *ppgk*) expression, they deleted *iolR* and restored cell growth in PTS deficient strains (Zhou et al., 2015). In addition, the *iolT1* transcript increased 5-fold, *iolT2* transcript increased 3-fold, *ppgk* and *glk* transcripts increased 2-fold in their deleted *iolR* strain. The glucose comsumption rate was also higher than the its parental (PTS deficient) strain, with rates of 2.68 and 1.49 mM/h, respectively. Therefore, we deleted *iolR* (Strain S5) and restored the cell growth to normal rates (Figure 2c). The S5 strain was able to produce 0.202 mg/mg of shikimate content and 1.37 g/L of shikimate titer after 48 h (Figures 2a,b). Shikimate content improved 2.9-fold compared to its parental strain.

### 3.4 *iolR* deletion increased *iolT2* and *glk* expressions during exponential growth phase in PTS deficient strain compared to PTS strain

**FIGURE 3:**
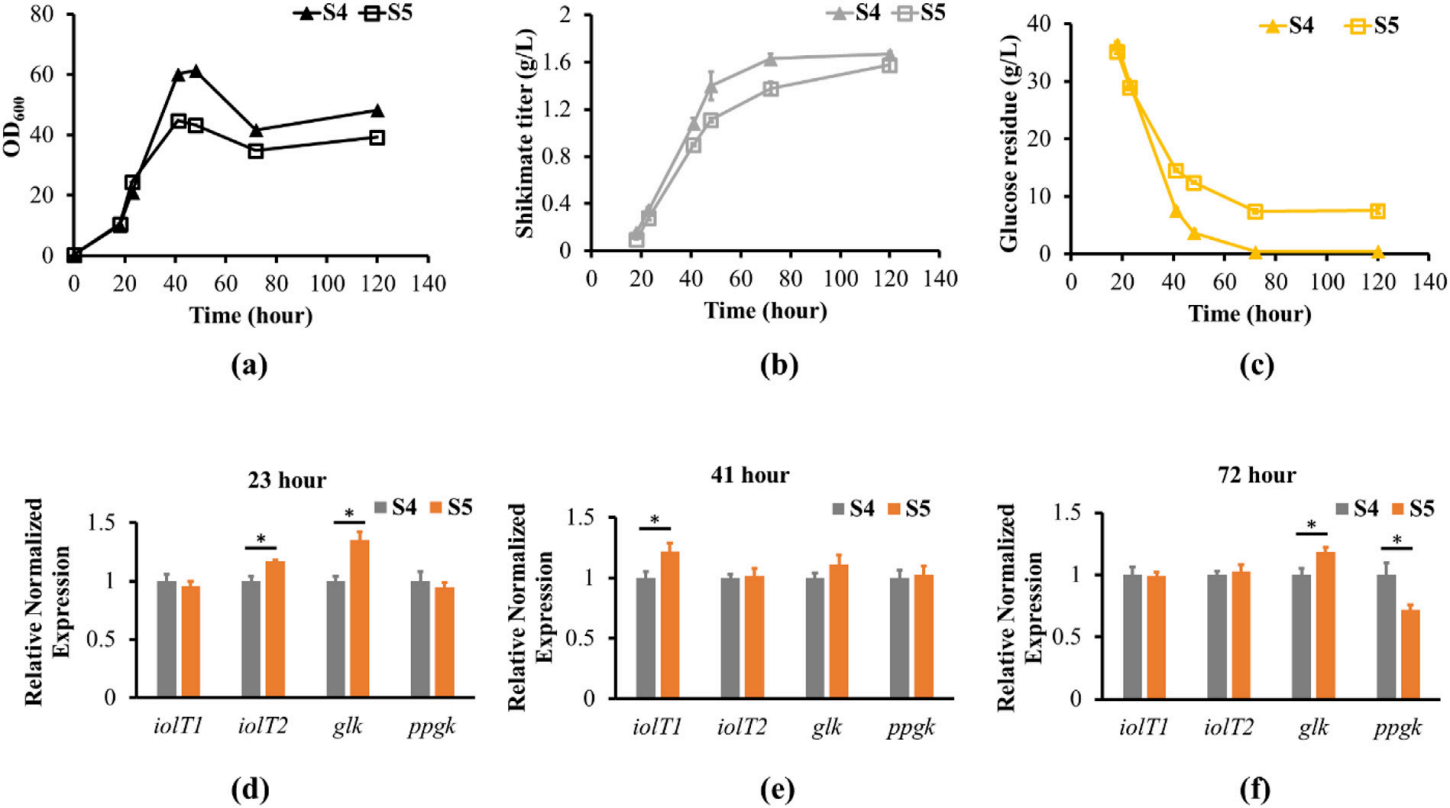
Shikimate, residual glucose, growth and transcript levels of engineered strains S4 and S5. **(a)** S4 and S5 strains were cultured in 250 mL shake flask with defined CGXII minimum medium supplemented with 4% glucose for 120 h. The cell growth curves of S4 (filled triangle) and S5 (opened square) are shown. **(b)** Shikimate titers (g/L) were measured at 18, 23, 41, 72 and 120 h for S4 (filled triangle) and S5 (opened square). **(c)** Glucose residue (g/L) was measured at 18, 23, 41, 72 and 120 h for S4 (filled triangle) and S5 (opened square) using HPLC with Aminex HPX-87H Ion Exclusion column. **(e,f)** Total RNA was prepared from S4 and S5 grown to 23, 41 and 72 h, corresponding to exponential, late exponential and stationary phases. The transcript levels of *iolT1*, *iolT2*, *glk* and *ppgk* from S4 (gray bar) and S5 (orange bar) were normalized to the reference gene (*leuA*). The transcript levels in S4 are set to 1. Data are averages and standard deviation error of the results from triplicates (* represent p-value<0.05 for two-tailed t-test).

To examine the efficiency of glucose uptake in the non-PTS with *iolR* deletion strain (S5) compared to the PTS strain (S4), residual glucose levels in the growth medium were determined up to 120 h (Figure 3c). As strain S5 grew to stationary phase at 72 h, 7.5 g/L of residual glucose remained in the culture medium, and did not get utilized even after 120 h of growth (Figures 3a,c). However, glucose was almost depleted at 72 h of growth for the PTS strain. The glucose uptake rate was slightly slower in the S5 strain than the S4 strain according to the residual glucose in the medium throughout the cell growths. The results suggested S5 was not able to uptake more glucose during stationary phase. Furthermore, we quantified the transcript levels of *iolT1*, *iolT2*, *glk* and *ppgk* during exponential, late-exponential and stationary phases to identify the potential causes of the lack of glucose uptake during stationary phases in *iolR* deletion strain. When the S4 and S5 strains reached exponential growth phase at 23 h, the *iolR* deletion strain (S5) had higher *glk* and *iolT2* transcript levels by 1.35-fold and 1.17-fold compared to S4 strain, respectively (Figure 3d). However, S5 did not have significant changes in *ppgk* and *iolT1* transcript levels compared to the S4 strain. The differences in expression levels across the genes were similar at late exponential phase (41 h), except S5 had higher *iolT1* transcript level by 1.22-fold compared to S4 strain (Figure 3e). At stationary growth phase (72 h), the *glk* transcript level increased by 1.18-fold, the *ppgk* transcript level reduced by 1.37-fold, and the *iolT1* and *iolT2* transcript levels were similar to the S4 strain (Figure 3f). In addition, the shikimate titer of S4 saturated at 72 h after glucose was depleted (Figure 3b). The shikimate titer of S5 continued to increase after 72 h, even though glucose uptake stopped. The cell morphologies of the S5 and S4 are similar during the late exponential growth phase at 41 h (Supplementary Figure S3a,b). When the cells were in stationary growth phase at 72 and 120 h, the S4 cells were forming more clusters than the S5 cells (Supplementary Figure S3c–f). It suggests the S4 is under certain stresses or limitation to promote cell growth at stationary growth phase. These results demonstrated increasing expression of *iolT1* or *iolT2 and glk* might contribute to cells’ ability to uptake glucose in the PTS deficient strain for improving cell growth.

### 3.5 Elimination of feedback inhibition to improve shikimate production

**FIGURE 4:**
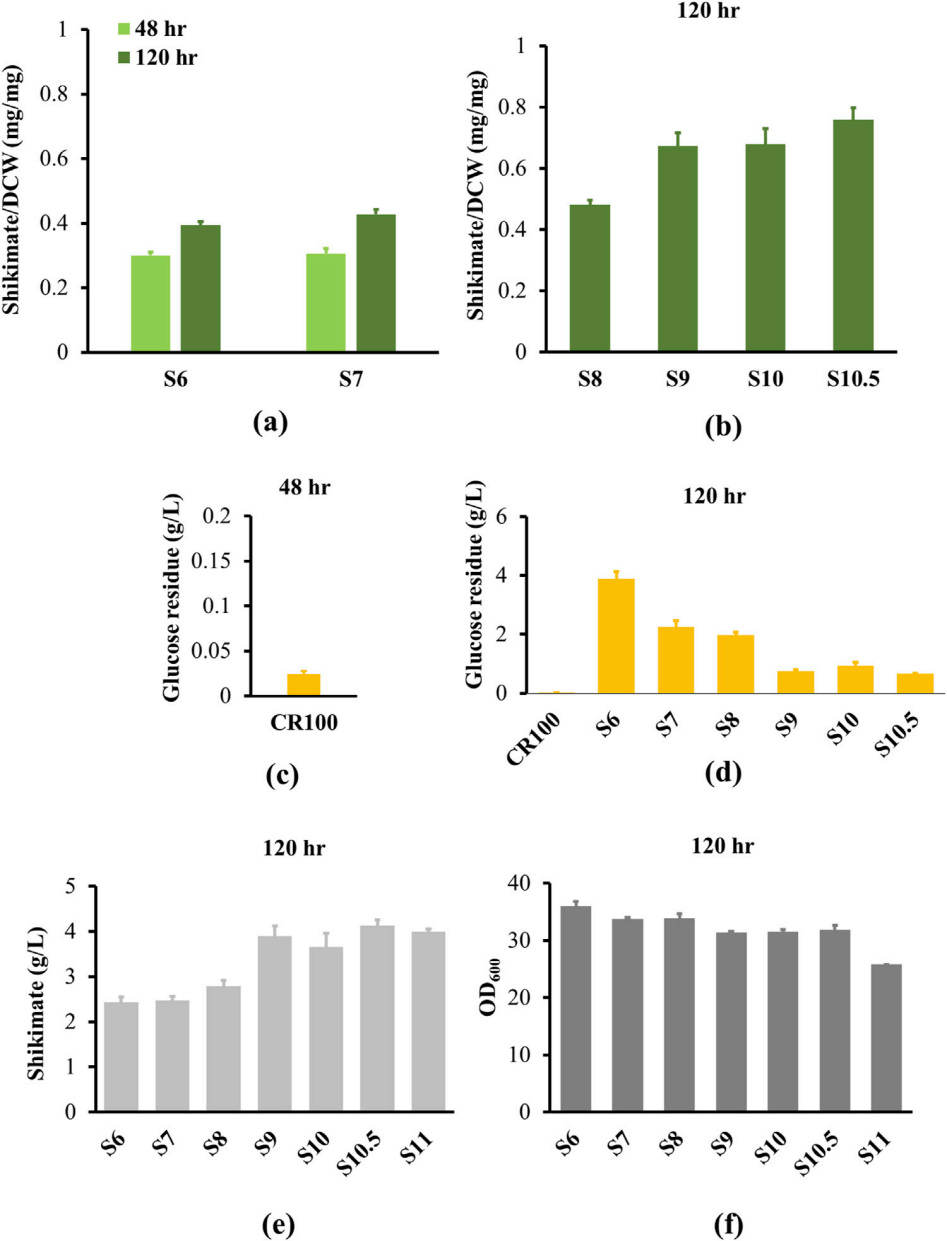
Shikimate and growth analysis from engineered strains. All strains were cultured in 250 mL shake flask with the define CGXII minimum medium supplemented with 4% glucose for 120 h. **(a)** Shikimate contents (mg/mg DCW) were measured at 48 h (light green bar) and 120 h (dark green bar) for S6 and S7. **(b)** S8, S9, S10 and S10.5 strains were measured at 120 h. **(c)** Glucose residue was measured for parental strain (CR100) at 48 h. **(d)** Glucose residue was measured for CR100 and S4 to S10.5 strains at 120 h. **(e)** Shikimate titers (g/L) were measured at 120 h for S6 to S11. **(f)** Comparison of cell growth of S6 to S11 strains. Data are averages and standard deviation error of the results from triplicates. OD_600_, optical density at 600 nm.

3-Deoxy-D-arobino-heptulosonic acid 7-phosphate (DAHP) is the precursor for shikimate production, which is synthesized by DAHP synthase (AroG_*E. coli*_) from PEP and erythrose-4-phosphate. AroG has been shown to be strongly inhibited by phenylalanine, but a single point mutation was identified in AroG of *E. coli* that eliminated phenylalanine feedback inhibition (Ger et al., 1994). The S180F mutation resulted in reduction of AroG inhibition from 60% to less than 10% in 20 mM phenylalanine. To eliminate feedback inhibition of AroG, we overexpressed the mutated *aroG*_*E. coli*_ with a strong constitutive promoter (*tac*) and replaced the native *aroG* in the S5 genome (strain S6). As a result, shikimate content in S6 reached 0.3 mg/mg after 48 h, and continued to accumulate to 0.39 mg/mg after 120 h due to a slower growth rate of strain S6 (Figure 4a; Supplementary Figure S4). Shikimate content improved 1.48-fold compared to its parental strain. The culture reached a final OD600 of 36 after 120 h, where the shikimate titer in S6 accumulated to 2.43 g/L (Figures 4e,f). However, there was still 3.9 g/L of glucose remaining in the culture medium after 120 h, while there was only 0.02 g/L of residual glucose detected in the culture medium after 48 h from the CR100 shikimate starter strain (Figures 4c,d). Therefore, additional strategies to direct glucose to shikimate must be introduced.

### 3.6 Improved glucose utilization and uptake for shikimate production

After feedback inhibition of AroG was eliminated, the genes encoding transketolase and transaldolase (*tkt* and *tal*) were overexpressed in an operon with a strong constitutive promoter (trc) (Strain S7) to improve shikimate production through the pentose phosphate pathway. The growth rates of S7 and S6 are similar (Supplementary Figure S3). The shikimate content in S7 was not statistically different from S6 after 48 h of growth (Figure 4a). After 120 h of growth, the shikimate content in S7 reached 0.42 mg/mg, equivalent to a 7.6% increase compared to S6 strain, while the shikimate titer in S7 accumulated to 2.47 g/L, equivalent to a 1.6% increase compared to the S6 strain (Figure 4e). There was still a substantial amount of residual glucose (2.25 g/L, 1.7-fold less than S6 parental strain) detected in the culture medium (Figure 4d), indicating that glucose import and/or utilization remains impaired. To determine whether the non-PTS pathway is the rate-limiting step, *iolT1* was overexpressed with a trc promoter to reinstate passive glucose transport (Strain S8). However, 1.98 g/L of glucose was still detected in the S8 culture medium, and shikimate content in S8 only reached 0.48 mg/mg and shikimate titer accumulated 2.79 g/L after 120 h (Figures 4b,d,e), suggesting that there is a bottleneck upstream of shikimate and downstream of glucose import.

### 3.7 Improving shikimate production via shikimate and glycolysis pathways

First, we improved the shikimate pathway immediately upstream of shikimate. The DHQ synthase (*aroB*), DHQ dehydratase (*aroD*) and shikimate dehydrogenase (*aroE*) are involved in the synthesis of shikimate (Figure 1b). Overexpression of *aroB*, *aroD* and *aroE* had been previously shown to significantly improve shikimate titer in *C. glutamicum* (Kogure et al., 2016)*.* We integrated heterogeneous genes of *aroB*, *aroD* and *aroE* from *Corynebacterium efficiens* in an operon and overexpressed with a strong constitutive promoter (*trc*) in the S8 genome (Strain S9). As a result, the shikimate content (0.67 mg/mg) and titer (3.9 g/L) increased 1.4-fold compared to S8 after 120 h (Figures 4b,e). The glucose remaining in the culture medium reduced 2.6-fold compared to the S8 strain (Figure 4d). The growth rate is similar between S8 and S9 (Figure 4f).

We then increased activity of the glycolysis enzyme to direct glucose to PEP, which would theoretically result in increased shikimate yield. Glucokinase (*glk*) and polyphosphate glucokinase (*ppgk*) phosphorylate the carbon at position 6 in glucose to form glucose-6-phosphate, eventually leading to the production of PEP. Therefore, we overexpressed *glk* and *ppgk C. efficiens* under a trc promoter in S9 genome (Strain 10). However, overexpression *of glk and ppgk* (S10) did not significantly improve shikimate content or titer compared to S9 (Figures 4b,e). Furthermore, glucose-6-phosphate is converted to fructose 1,6-bisphosphate (FBP). FBP is then converted to dihydroxyacetone phosphate (DHAP) by Fructose-bisphosphate aldolase (*fba*). Fructose-bisphosphate aldolase has a reversible function of producing DHAP back to FBP. Overexpression of *fba*_*Elaeis guineensis*_ has also been previously demonstrated to increase downstream product (lipid content) in *Saccharomyces cerevisiae* (Ruzlan et al., 2017). Furthermore, Glyceraldehyde-3-phosphate dehydrogenase (*gapDH*) converts Glyceraldehyde 3-phosphate (GAP) to 1,3-disphosphoglyceric acid (BPG), a precursor to PEP required for shikimate production. We then overexpressed *fba* and *gapDH* from *E. guineensis* under a *tac* promoter in the pZ8 plasmid (Strain S10.5), which increased shikimate content to 0.76 mg/mg (Figure 4b) and produced 4.1 g/L (Figure 4e) of shikimate titer after 120 h (0.13 g/g, 0.034 g/L/h). This is equivalent to a 12% increase in shikimate content compared to its parental strain S10. The strains S10 and S10.5 have similar growth rates (Figure 4f). To create a more stable strain, we integrated the *fba* and *gapDH* operon in strain S10 (Strain 11). The final strain S11 produced 3.99 g/L of shikimate after 120 h (Figure 4e).

## 4 Discussion

Production of shikimate in *C. glutamicum* R has been of interest over the last few years. An impressive shikimate titer of 141 g/L of shikimate was produced with a 51% (mol/mol) shikimate yield from the glucose consumed (Kogure et al., 2016). Based on Kogure *et al.*,‘s metabolic engineering strategies, they utilized the non-PTS pathway to direct more carbon flux to shikimate. However, the non-PTS pathway reduced cell growth. To restore cell growth, the myo-inositol transporter (*iolT1*) and three glucokinases (*glk1*, *glk2* and *ppgk*) for glucose uptake and phosphorylation were constitutively expressed. In recent years, another strategy was explored to restore growth of *C. glutamicum* using the non-PTS pathway to improve succinic acid yield (Zhou et al., 2015). The strategy comprised of deleting *iolR*. After they deleted *iolR* in their PTS deficient strain, the cell growth and glucose consumption were restored. Furthermore, they overexpressed *iolT1* and *ppgk* in their deleted *iolR* strain. This strain had similar cell growth and glucose consumption to their wild type strain and improved succinic acid yield by 50%. In this study, we produced shikimate in a PTS deficient strain with the elimination of *iolR*. Further, we improved shikimate production through metabolic engineering. According to our result, the deletion of *aroK*, *qsuD*, *qsuB*, overexpression of *aroG* ^*S180F*^ and *aroBDE* genes together with the deletion of *iolR* have the most impact in improving the shikimate content in a PTS deficient strain. We were able to achieve 4.1 g/L of shikimate in shake flask.

After deletion of *aroK* and *qsuD*, shikimate was able to accumulate while no carbon flux was directed to aromatic amino acids production. Therefore, it required a supplement of 100 mg/L of each aromatic amino acids and 50 mg/L *p*-aminobenzoate to restore cell growth, which substantially raised the cost of shikimate production. To eliminate the need to add aromatic amino acids and *p*-aminobenzoate, a genetic switch is needed to control *aroK* expression. It is beyond the scope of this manuscript and required further investigation.

In recent years, many groups have achieved shikimate overproduction in various microorganisms, such as *E. coli* and *C. glutamicum.* In *E. coli*, they were able to achieve 126.4 g/L of shikimate in fed-batch bioreactor and achieve 0.5 g/g shikimate yield from glucose (Li et al., 2023). In *C. glutamicum R,* they were able to achieve 141 g/L of shikimate in growth arrest fermentation and achieve 51% (mol/mol) shikimate yield from glucose after 48 h (Kogure et al., 2016). In the current study, the final *C. glutamicum* strain S10.5 only achieved 4.1 g/L of shikimate with 13% (mol/mol) shikimate yield from glucose after 120 h in shake flasks. The lower shikimate yield could be due to utilization of the non-PTS pathway and lower efficiency in directing carbon flux from glucose to the shikimate pathway. The results show that constitutively expressed endogenous *iolT1* and glucokinases restore glucose utilization more efficiently in non-PTS *C. glutamicum* strains than the deletion of *iolR*. Interestingly, deletion of *iolR* has shown to increase expression of *iolT1 or iolT2* and glucokinase during the exponential and late exponential growth phases, but *iolT1* and *iolT2* was unchanged during the stationary growth phase in PTS deficient strain compared to PTS strains. Our result demonstrates after deletion of *iolR* in the PTS deficient strain, the growth rate, shikimate titer, and glucose uptake rate were similar to PTS strain during the exponential growth phase, where *iolT2* and *glk* expression increased by 17% and 35% in the PTS deficient strain, respectively. Perhaps, increasing expressions of *iolT2* and *glk* might have synergistic effect in restoring cell growth and glucose uptake completely in PTS deficient strain with *iolR* deleted. During the late-exponential growth phase, the *iolT1* expression was increased, but *iolT2* expression was similar in the PTS deficient strain (S5) and PTS strain (S4). In addition, the growth and glucose uptake rates of S5 were slower than S4 strain. The expression of *iolT2* and *glk* could possibly play an important role in glucose uptake in PTS deficient strains for downstream products formation and cell growth. Further experiment will be needed to investigate the overexpression of *iolT2* and *glk* in PTS deficient strains.

Our shikimate yield is 3.9-fold less compared to Kogure et al.,’s. In addition, our shake flask experiment might also yield lower shikimate under high cell density growth arrest fermentation in bioreactor. Kogure et al., did their experiment in high cell density growth arrest fermentation, it required 10% cells (w/v) in the initial inoculation. Further investigation is needed with our best shikimate strain under high cell density growth arrest fermentation conditions. Challenges remain in our engineered shikimate producing strain with respect to the yields achieved during biosynthesis of shikimate relative to the maximum yields of shikimate that can be biosynthesized from glucose. Alternatively, integration of a glucose facilitator and a glucokinase from *Z. mobilis* in the non-PTS strain in *C. glutamicum* is a potential strategy to enhance shikimate production.

## Data Availability

The raw data supporting the conclusions of this article will be made available by the authors, without undue reservation.
