# Supplementary material for: The effect on shikimate production by deleting iolR and metabolic engineering in PTS deficient Corynebacterium glutamicum strain
Source: Front Bioeng Biotechnol. 2025 Jun 26;13:1616558. doi: 10.3389/fbioe.2025.1616558 (PMC12241104; doi:10.3389/fbioe.2025.1616558)
Supplement: Supplementary file 1 [file DataSheet1.docx]

**Supplementary Material**

**1 Supplementary Figures and Tables**

- 1. **Supplementary Figures**

**
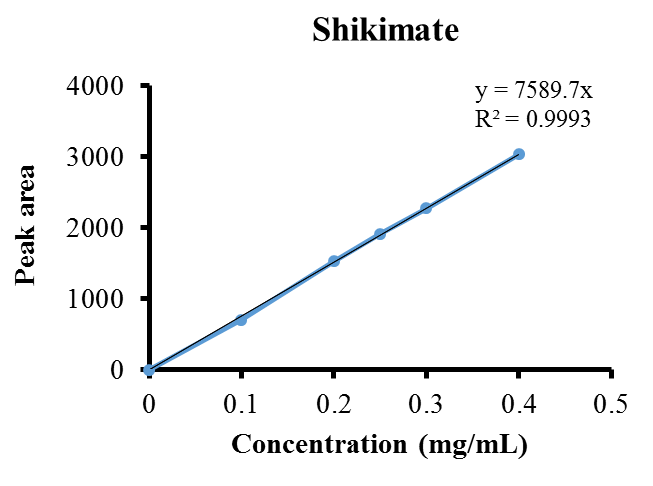
**

**Figure S1.** HPLC analysis of shikimate standard. Different shikimate concentrations were measured by HPLC at 210 nm to obtain a shikimate standard curve.

**
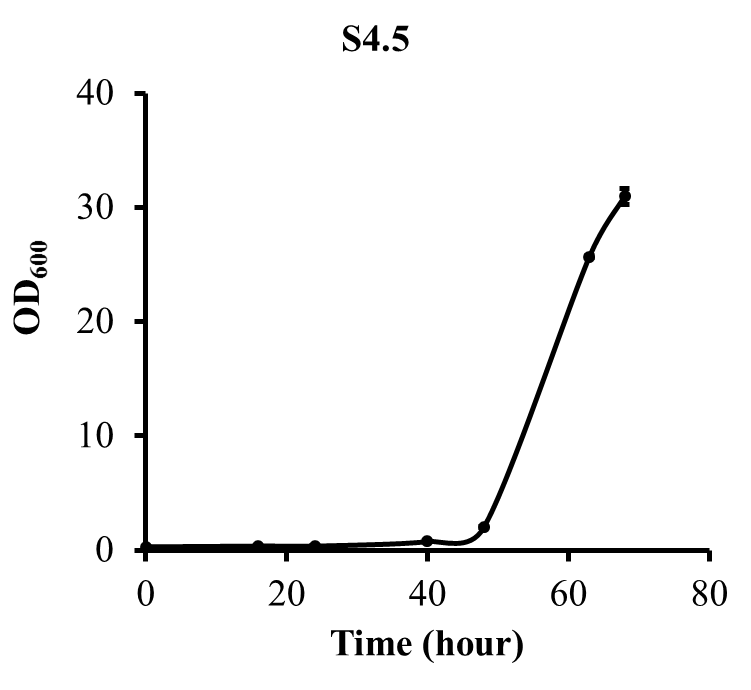
**

**Figure S2:** Growth curve of engineered strains S4.5. To observe the phenotypes after gene deletion and insertion, strains were cultivated in the define CGXII minimal medium with 4% glucose as the only source of carbon and energy. The cell growth curves of S4.5 (black line) is shown. Data are averages standard deviation error of the results from triplicates. OD_600_, optical density at 600 nm.

**
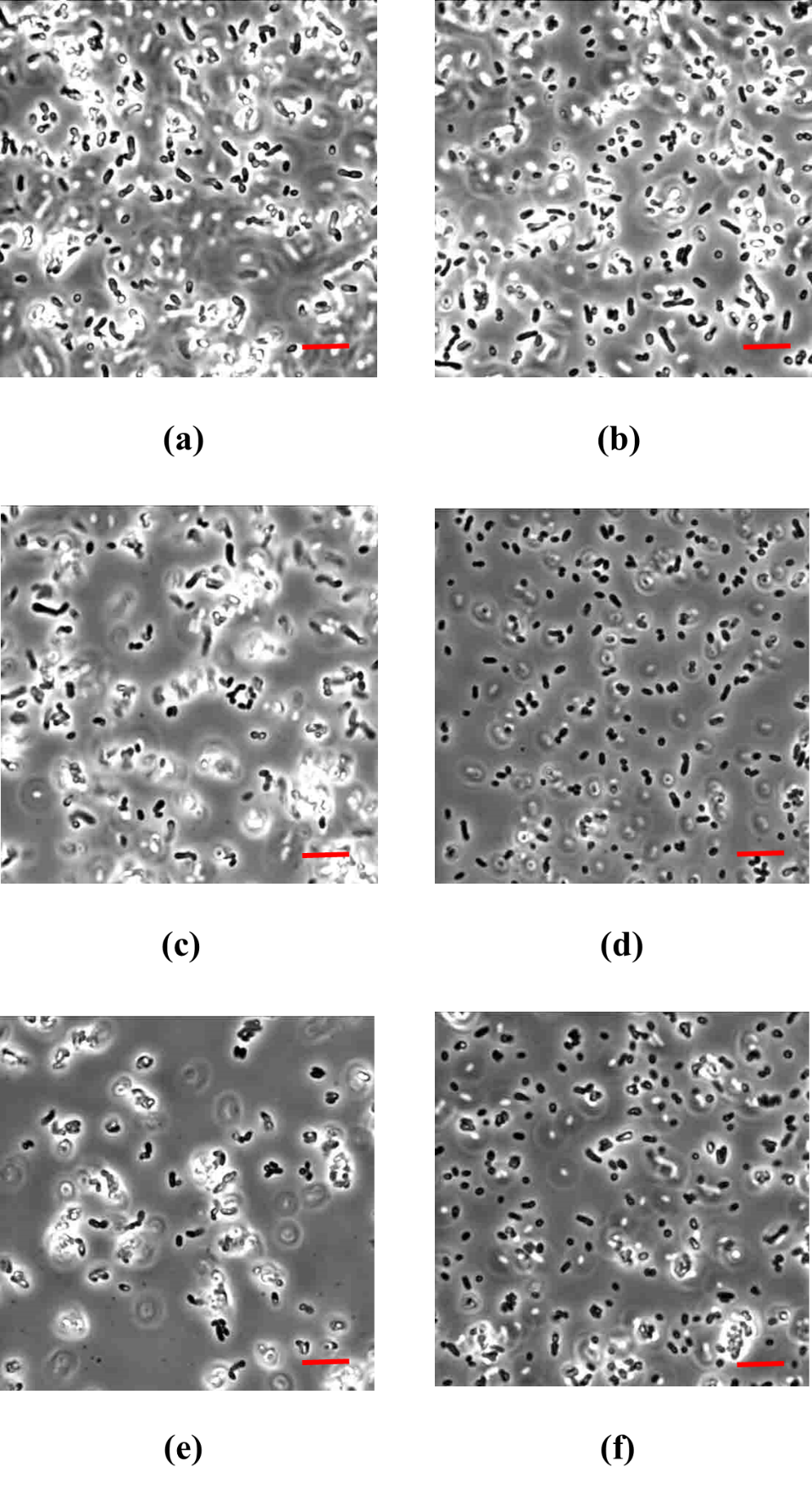
**

**Figure S3:** The morphology of S4 and S5 cells. The S4 strain was grown to 41 hour (a), 72 hour (c) and 120 hour (e), and viewed under microscope at 100X. The S5 strain was grown to 41 hour (b), 72 hour (d) and 120 hour (f), and viewed under microscope at 100X. The red line represents 10 micrometer.

**
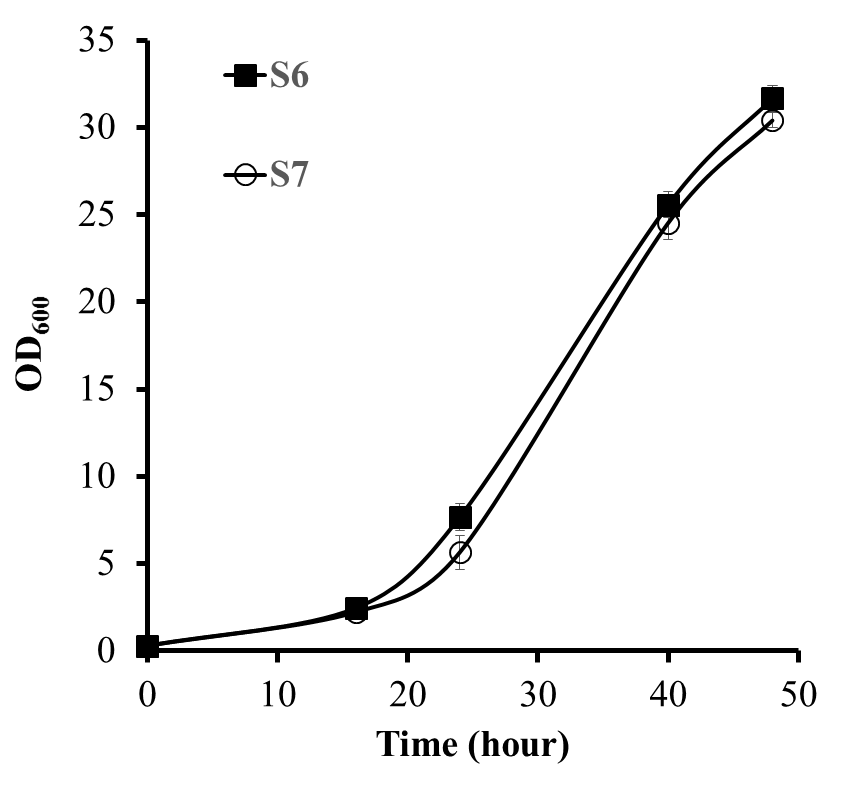
**

**Figure S4:** Growth curve of engineered strains S6 and S7. To observe the phenotypes after gene modifications, strains were cultivated in the defined CGXII minimal medium with 4% glucose as the only source of carbon and energy. The cell growth curves of S6 (filled square) and S7 (open diamond) are shown. Data are mean and standard deviation error of the results from triplicates. OD_600_, optical density at 600 nm.

- 1. **Supplementary Tables**

**Table S1.** Bacterial plasmids used in this study.

| **Plasmids** | **Characteristics** | **Source** |
| --- | --- | --- |
| pK18mobsacB | KmR, sacB, mobilizable *E. coli* vector used for construction of deletion and integration in *C. glutamicum* | Schafer et al. (1994) |
| pZ8-1 | *E. coli - C. glutamicum* shuttle expression vector | Dusch et al. (1999) |
| pK18daroK | pK18mobsacB derivative for the markerless deletion of *aroK* gene in *C. glutamicum* | This work |
| pK18dqsuD | pK18mobsacB derivative for the markerless deletion of *qsuD* gene in *C. glutamicum* | This work |
| pK18dqsuB | pK18mobsacB derivative for the markerless deletion of *qsuB* gene in *C. glutamicum* | This work |
| pK18dldh | pK18mobsacB derivative for the markerless deletion of *ldh* gene in *C. glutamicum* | This work |
| pK18dhdpA | pK18mobsacB derivative for the markerless deletion of *hdpA* gene in *C. glutamicum* | This work |
| pK18dptsH | pK18mobsacB derivative for the markerless deletion of *ptsH* gene in *C. glutamicum* | This work |
| pK18diolR | pK18mobsacB derivative for the markerless deletion of *iolR* gene in *C. glutamicum* | This work |
| pK18aroGaroGS180F | pK18mobsacB derivative for replace *aroG* with overexpressed *aroG^S180F^* *E. coli* in *C. glutamicum* | This work |
| pK18trctkt | pK18mobsacB derivative for integrate a strong constitutive promoter (trc) in front of *tkt* and *tal* operon in *C. glutamicum* | This work |
| pK18trciolT1 | pK18mobsacB derivative for integrate a strong constitutive promoter (trc) in front of *iolT1* in *C. glutamicum* | This work |
| pK18dldharoDE | pK18mobsacB derivative for replace *ldh* with overexpressed *aroD* and *aroE* from *C. efficiens* in *C. glutamicum* | This work |
| pK18aroB | pK18mobsacB derivative for integrate *aroB*_C. efficiens_ after aroE*_C. efficiens_* in *C. glutamicum* | This work |
| pK18glkppgk | pK18mobsacB derivative for integrate *glk* and *ppgk* after *iolT1* in *C. glutamicum* | This work |
| pK18fbagapDH | pK18mobsacB derivative for integrate *fba* and *gapDH* after *aroB_C. efficiens_* in *C. glutamicum* | This work |
| pZ8fbagapDH | pZ8-1 containing overexpress *fba* and *gapDH* from oil palm under tac promoter in multiple copy plasmid | This work |
|  |  |  |

**Table S2.** Primers used in this study.

| **Primer names** | **Primer sequences** | **Plasmid construction** |
| --- | --- | --- |
| AROBFOR | TATGACCATGATTACGAATTCACCGCAGCGTCCACCATGG | pK18aroB |
| AROBREV | TTAAACCGGGCACCTGATTAAC |  |
| AROCFOR | TAATCAGGTGCCCGGTTTAATTCATTACGCTCCATCCTC |  |
| AROCREV | CAGGTCGACTCTAGAGGATCTTCTGGCGAAGATCGCCTCG |  |
| QSUANEWFOR | TATGACCATGATTACGAATTATTGGTCGCCGACCAGTGTTC | pK18dqsuB |
| QSUANEWREV | GTGGTCAAAGATTCCTTTTGGTTTTACTGAG |  |
| ARODNEWFOR | CAAAAGGAATCTTTGACCACCATCCCGAACTAGCCCCCCAAC |  |
| ARODNEWREV | CAGGTCGACTCTAGAGGATCTCATATTTTTGGGCTTTCTACTTTTTGAGATTTGC |  |
| ACPMFOR | TATGACCATGATTACGAATTTTTAGACCCGGGGTACGGTTT | pK18dhdpA |
| CG2472REV | ATGCCATGACCTACAGAATAAACACCATTGTCCCTGTTTTGGG |  |
| CG2475FOR2 | TATTCTGTAGGTCATGGCATTTGCAGAC |  |
| CG2475REV | CAGGTCGACTCTAGAGGATCCTCGGGGAAGCCAGGTGAC |  |
| PTSFFOR | TATGACCATGATTACGAATTCCTGGCATTCGTCTCCGAAG | pK18dptsH |
| PTSFREV | GGAAAGTGTCCTTTCGTGTTGCTAC |  |
| CG2124FOR | AACACGAAAGGACACTTTCCACAACGCTCTGCTTGTTAAAAGC |  |
| CG2124REV | CAGGTCGACTCTAGAGGATCACGAGGTTAAGGGAATTAACCCGAG |  |
| CG195FOR | TATGACCATGATTACGAATTCGCTTCTTGACGCCCCCA | pK18diolR |
| CG195REV | GAAATAAACCAAAGAGCCCTTCTGAACTG |  |
| IOLCFOR | AGGGCTCTTTGGTTTATTTCGATGTCTCCTTTCGTTGCCCAC |  |
| IOLCREV | CAGGTCGACTCTAGAGGATCTATCTACGCCCAAGCGGCC |  |
| CG2392FOR | TATGACCATGATTACGAATTACGCAGTTAGCACACAGTTTGATTC |  |
| CG2392REV | CCACACATTATACGAGCCGGATGATTAATTGTCAAACCCCTATTCATAGCACGATATCGG |  |
| AROGFOR | CCGGCTCGTATAATGTGTGGAAAAGGAGGACAACCATGAATTATCAGAACGACGATTTAC | pK18aroGaroGS180F |
| AROGS180FREV | TTTTTGAAGCCGACCGGACAAAAAAGCCCTGATGCCAGTTC |  |
| AROGS180FFOR | TGTCCGGTCGGCTTCAAAAATG |  |
| AROGREV | TTTAGAGTTGGTCTAAAAGCTTACCCGCGACGCGCTTTTAC |  |
| CG2390FOR | GCTTTTAGACCAACTCTAAAAATAGGTGTGG |  |
| CG2390REV | CAGGTCGACTCTAGAGGATCTCCTCACTGCCCACCATCTTG |  |
| CTABFOR | TATGACCATGATTACGAATTAAAACTCGCGCGGAACCAG | pK18trctkt |
| CTABREV | CCACACATTATACGAGCCGGATGATTAATTGTCAATGGGTTAAACCGGGACCAAAG |  |
| TKTTRCFOR | CCGGCTCGTATAATGTGTGGAAAAGGAGGACAACCATGACCACCTTGACGCTGTCAC |  |
| TKTTRCREV | CAGGTCGACTCTAGAGGATCTCGCCCTCAGCAGCGGTTG |  |
| IOLT1FOR | CCGGCTCGTATAATGTGTGGAAAAGGAGGACAACCATGGCTAGTACCTTCATTCAGGCC | pK18trciolT1 |
| IOLT1REV | CAGGTCGACTCTAGAGGATCCCAACAACAATCATCAACTCATTACGGC |  |
| CG228REV | CCACACATTATACGAGCCGGATGATTAATTGTCAAAAGTTTGTCGTGCCCTTACTTGG |  |
| CG228FOR | TATGACCATGATTACGAATTCGGTCGATCGACGTCTCGAG |  |
| CG3220FOR | TATGACCATGATTACGAATTAAAACAGCCAGGTTAGCAGC |  |
| CG3220REV | CACACATTATACGAGCCGGATGATTAATTGTCAATTTCGATCCCACTTCCTGATTTC |  |
| ARODFOR2 | TCCGGCTCGTATAATGTGTGGAAAAGGAGGACAACCATGCATGTTCTCGTGATCAACGG | pK18dldharoDE |
| ARODREV | ATGGTTGTCCTCCTTTCTAGCGCTCCCGATCGGCGAG |  |
| AROEFOR | CTAGAAAGGAGGACAACCATGACTGACAAACCCACCCAC |  |
| AROEREV | TCACATACCCAGTGAGGCC |  |
| AROECENEWFOR4 | TATGACCATGATTACGAATTCTGGTCGAATTGCTCGGCG |  |
| AROECEREV4 | ATGGTTGTCCTCCTTTTCACATACCCAGTGAGGCC |  |
| AROBCENEWFOR4 | GTGAAAAGGAGGACAACCATGCACGTGACCACCATCTTCGAT |  |
| AROBCENEWREV4 | CTAGCTGCTGATGGCCTCGTA |  |
| CG3218NEWFOR4 | ACGAGGCCATCAGCAGCTAGATCTTTGGCGCCTAGTTGG |  |
| CG3218REV | CAGGTCGACTCTAGAGGATCGAGAATTTCGGCGTGCTCG |  |
| CG0223NEWFOR | TATGACCATGATTACGAATTGTGCATTCATCGCACTGTGGATG |  |
| CG0223NEWREV | TGACATGGTTGTCCTCCTTTTTAGTGCACCTTTCCTTTTCGGATG |  |
| GLKCEFOR | AAAGGAGGACAACCATGTCACAAGACCCTGACCGTTTC | pK18glkppgk |
| GLKCEREV | GGTTGTCCTCCTTTTCAGGGTGCCGCGACCGC |  |
| PPGKCEFOR | CCCTGAAAAGGAGGACAACCATGACTGAAACGGGATTTGGAATCG |  |
| PPGKCEREV | TCTATGCAGTGTCTGGGTTTCTAGGGGGCCAGGTGTTTTG |  |
| CG0222NEWFOR | AAACCCAGACACTGCATAGATAACAC |  |
| CG0222NEWREV | CAGGTCGACTCTAGAGGATCTGACAGTTCTTGGACTGGTGGC |  |
| PK18MOBSACBFOR | GATCCTCTAGAGTCGACCTG |  |
| PK18MOBSACBREV | AATTCGTAATCATGGTCATAG |  |
| EGFBARUIFOR | TTCACACAGGAAACAGAATTCCCGGGAAAGGAGGACAACCATGGCATCCGCAACCTTGC | pK18fbagapDH |
| EGFBARUIREV | GGTTGTCCTCCTTTTTAGTAGGAGTAGTTCTTGACGTACATGCC |  |
| EGGAPDHNEWF | TACTAAAAAGGAGGACAACCATGTTCGTGGTGGGCGTG |  |
| EGGAPDHNEWR | AACAGCCAAGCTTGGCTGCAGGTCGACGTTACTGGGTGCCGTGGATGTG |  |
| GAPDHCG3218F | ACATCCACGGCACCCAGTAAATCTTTGGCGCCTAGTTGGC |  |

**Table S3.** DNA sequence of the *C. glutamicum* codon optimized genes.

| *fba Elaeis guineensis*  ATGGCATCCGCAACCTTGCTGAAGTCCTCCTTCCTGCCAAAGAAATCCGAATGGCTGAACGCACGCCCATCCTCCACCTCTCAGCCAATGGCAGTGTCCTTCACCGTTCGCGCAGGCGCATACGCAGATGAACTGGTCAAGACCGCAAAGACCGTGGCATCCCCAGGTCGCGGTATCCTGGCAATGGATGAATCCAACGCAACCTGCGGCAAGCGCCTGGCATCCATCGGCCTGGAAAACACCGAAGCAAACCGCCAGGCCTACCGCACCTTGCTCATCACCGCACCAGGCCTCGGCCAGTACGTGTCCGGTGCAATCCTGTTCGAAGAAACCCTGTACCAGTCCACCACCGATGGCAAGAAAATGGTGGATGTGCTGGTGTCCCAGAACATCATGCCAGGCATCAAGGTGGATAAGGGCCTCGTGCCACTGGCAGGCTCCAACGATGAATCCTGGTGCCAGGGCCTCGATGGCCTGGCTTCCCGCTGCGCTGCATACTACCAGCAGGGTGCACGCTTCGCAAAGTGGCGCACCGTGGTGTCCATTCCAAACGGTCCATCCGCACTGGCAGTGAAAGAAGCAGCCTGGGGACTCGCACGCTACGCAGCAATCGCACAGGATAACGGCCTGGTTCCAATCGTGGAACCAGAAATCCTGCTGGATGGCGATCACGGCATCGAACGCACCTTCGAAGTGGCACAGAAAGTGTGGGCTGAAGTGTTCTTCTACATGGCAGAAAACAACGTGATGTACGAAGGCATCCTGCTGAAGCCATCCATGGTGACCCCAGGCGCAGAATGCAAAGAACGCGCAACCCCAGAACAGGTGGCAGAATACACCCTGAAGCTGCTGCACCGTCGCATCCCTCCAGCAGTGCCAGGTATCATGTTCCTGTCCGGTGGCCAGTCCGAAGTGGAAGCAACCCTGAACCTGAACGCAATGAACCAGTCTCCACATCCATGGCACGTGTCTTTCAGCTTCGCACGCGCACTGCAGAACACCTGTCTCAAGACCTGGGGTGGTCGCCCAGAAAACGTGAAGGCAGCACAGGATGCACTGCTGATTCGCGCAAAGGCAAACTCCCTGGCTCAGCTGGGCAAGTACACCGGTGAAGGCGAATCCGCTGAAGCAAAAGAAGGCATGTACGTCAAGAACTACTCCTACTAA  *gapDH Elaeis guineensis*  ATGTTCGTGGTGGGCGTGAACGAAAAAGAATACAAGTCCGATATCAACGTGGTGTCCAACGCATCCTGCACCACCAACTGCCTGGCACCACTGGCAAAGGTGATCCACGATAAGTTCGGCATCGTGGAAGGCCTGATGACCACCGTGCACTCCATCACCGCAACTCAGAAAACCGTGGATGGCCCATCCTCCAAGGATTGGCGTGGCGGTCGCGCAGCATCCTTCAACATCATCCCGTCCTCCACCGGTGCAGCAAAGGCAGTCGGCAAGGTGCTGCCAGCACTGAACGGCAAGCTGACCGGCATGGCATTCCGCGTGCCAACCGTGGACGTGTCCGTGGTGGATCTGACCGTGCGCCTGGAAAAGTCCGCAACCTACGATCAGATCAAGGATGCAATCAAAGAAGAATCCGAAGGCAAGATGAAGGGCATCCTGGGCTACGTGGATGAAGATCTGGTGTCCACCGATTTCTTGGGCGATTCCCGCTCCTCCATCTTCGATGCCAAGGCAGGTATCGCCCTCAACGGCAACTTCGTGAAGCTGGTGTCCTGGTACGATAACGAATGGGGCTACTCCTCTCGCGTGGTGGACCTCATCCGCCACATCCACGGCACCCAGTAA |
| --- |

**Institutional Review Board Statement:** Not applicable

**Informed Consent Statement:** Not applicable
